# Chronic Widespread Pain and Fibromyalgia Syndrome: Life-Course Risk Markers in Young People

**DOI:** 10.1155/2019/6584753

**Published:** 2019-05-05

**Authors:** Aidan C. Tan, Tiina Jaaniste, David Champion

**Affiliations:** ^1^Faculty of Medicine, University of New South Wales, Sydney, New South Wales, Australia; ^2^Department of Pain and Palliative Care, Sydney Children's Hospital, Sydney, New South Wales, Australia; ^3^School of Women's and Children's Health, University of New South Wales, Sydney, New South Wales, Australia

## Abstract

Although the life-course concept of risk markers as potential etiological influences is well established in epidemiology, it has not featured in academic publications or clinical practice in the context of chronic widespread pain (CWP) and fibromyalgia syndrome (FMS). Studies of risk markers are required considerations for evaluation of patients and for research because there is no single cause, pathological feature, laboratory finding, or biomarker for CWP or FMS. The early-life risk markers identified by extensive literature review with best evidence for potential causal influence on the development and progression of CWP and FMS include genetic factors, premature birth, female sex, early childhood adversity, cognitive and psychosocial influences, impaired sleep, primary pain disorders, multiregional pain, physical trauma, infectious illness, obesity and inactivity, hypermobility of joints, iron deficiency, and small-fiber polyneuropathy. The case history illustrates the potential etiological influence of multiple risk markers offset by personal resilience.

## 1. Introduction

The causal influences on persistent multisite, multiregional, or widespread pain disorders in a young person are often perplexing to physicians and patients. Despite evidence of abnormal nervous system findings, neurophysiological signatures, low-grade systemic inflammation and neuroinflammation, and biochemical alterations in plasma and muscle proteins [1–3], there are no sensitive and specific laboratory findings, biomarkers, or pathological features for chronic widespread pain (CWP) and fibromyalgia syndrome (FMS). Thus, in the absence of a single cause, the etiological focus shifts towards risk markers, defined by Burt [4], as attributes or exposures that are associated with an increased probability of disease, but do not necessarily constitute a causal factor. The conservative term risk marker is preferred to risk factor, association, or comorbidity in this context. Few risk markers for CWP and FMS have been empirically demonstrated to be causal, and there is uncertainty in the literature about whether a risk factor should be a causal link in the etiological chain or may be peripherally associated with an outcome. There is also uncertainty in the literature about what strength of association is needed for an attribute or exposure to be a risk factor for an outcome and how directly it needs to be associated with the outcome.

In this review, the risk markers of CWP and FMS in a young person were considered in a clinical vignette and from a life-course perspective. The life-course approach has demonstrated the influence of early-life events across various health outcomes, including pain [5, 6]. Identifying risk markers enables a shared understanding of the biopsychosocial antecedents of CWP and FMS and guides early identification, risk prediction, and targeted prevention. Ultimately, the hope is that a life trajectory towards chronic pain can be prevented or interrupted.

In young children, the most common pain disorders without overt disease or trauma—termed idiopathic, nonspecific, functional, or primary—are growing pains, recurrent abdominal pain, and headaches [7]. By age 14, the most common pain disorders are headache, abdominal pain, and back pain [8], and children with one pain disorder not infrequently experience multiple other pain disorders [9–15]. These childhood pain disorders are antecedents of chronic pain in adolescence and adulthood, with long-term persistence, particularly of recurrent abdominal pain and low back pain [16]. Predictors of the transition from acute to persistent musculoskeletal pain in children and adolescents in a prospective study are female sex and poorer conditioned pain modulation, while higher depressive symptoms are associated with higher pain-related disability and poorer quality of life [17]. These features, especially collectively, are likely antecedents to CWP.

CWP is defined as pain in upper and lower and right and left quadrants, for more than three months duration [18]. The prevalence of widespread pain in school-aged children is 7–15% [19, 20]. The prevalence of chronic multisite pain in female and male adolescents aged 13–19 is 11.4% and 3.8%, respectively [21], and in young adults aged 22 is 10.9% [22]. A twin study by Kato et al. found the overall prevalence of CWP to be 4.1%.

In CWP meeting criteria for FMS, cognitive, affective, and sensory features emerge, with evidence of abnormal brain networks, altered biology of pain processing, and disturbance of neurobiological function. FMS is defined, especially for epidemiological studies, by the presence of CWP accompanied by chronic fatigue, disturbed sleep, and a spectrum of symptoms associated with nociceptive amplification of neural signaling within the central nervous system [14, 23–27]. There are no validated criteria or evidence-based consensus guidelines for the diagnosis of juvenile FMS [28–30]. Ting et al. [31] concluded that the 2010/2011 American College of Rheumatology fibromyalgia criteria [25] had diagnostic applicability in adolescent females; however, there are no published studies of juvenile FMS which have applied the 2016 revisions to the 2010/2011 fibromyalgia criteria [29].

The onset of juvenile FMS is typically in late childhood and early adolescence. The prevalence of juvenile FMS in children and adolescents is estimated at 1.3%, but up to 3.5% to 6.2% for those aged between 15 and 19, and is substantially higher in females than males [32]. Of adolescents with juvenile-onset FMS, more than 80% demonstrate persistence of pain, physical impairment, emotional impairment, and other symptoms into early adulthood, and half later meet the full criteria for adult FMS [33].

CWP and FMS cases, as defined, lie on a spectrum from primary pain disorders [34] where the recently introduced term nociplastic [35] may be applied, to secondary disorders where a substantial causal risk factor or causal influence is evident, such as injuries in a motor vehicle accident. In some cases, termed primary FMS, there are no obvious peripheral nociceptive generators or life-course risk markers. In such cases, genetic factors and impaired endogenous pain modulation might apply, and primary pain disorders may precede or accompany the FMS. In other cases, risk markers are prominent, as in the clinical vignette.

### 1.1. Clinical Vignette

At the time of presentation, the patient, a 25-year-old woman employed in a clerical position, reported a history of persistent pains in wide distribution including hands, feet, ankles, knees, hip regions, back, and neck, as well as headaches. She could be “sore” all day, though was typically better in the morning and worse in the evening. The associated symptoms included her feet “going to sleep,” coldness and bluish-white discoloration of fingers and toes, fine tremor of hands, minor subjective swelling of hands, minor sleep impairment, waking unrefreshed, self-reported mild cognitive difficulties, and low energy. Her self-assessed mood was “okay,” but she acknowledged “a lot of anxiety” with a tendency to panic reactions. The pain had gradually evolved from childhood and had intensified during adolescence. No obvious disease had been identified, and extensive medical consultations and investigations for inflammatory rheumatic disorders had been negative or inconclusive.

The principal findings on examination were mild obesity, fine hand tremor, moderate hypermobility of joints (meeting Beighton score and arthralgia criteria), and widespread deep tenderness (low-pressure pain threshold). On cutaneous somatosensory testing at a time of relative quiescence of symptoms, responses to cutaneous stimuli, including static and repetitive punctate pressure, were normal. Responses to repetitive deep pressure stimuli at the pain threshold, in wide distribution, demonstrated facilitated temporal summation of pain intensity, minor persistence of after-sensations, but no radiation, interpreted as inferring mild generalized central sensitization. The 2016 revised diagnostic criteria for FMS [25–27] were met.

Past medical and family history revealed a remarkable life-course of potential vulnerability factors for chronic pain disorders:

- Family history of depression in both parents, significant anxiety in her father, and migraine in her maternal grandfather.
- Born six weeks premature.
- Childhood adversity through parental disharmony and subsequent divorce at 8 years of age.
- Anxiety and depression from childhood. At the time of presentation, anxiety was more prominent than depression, with occasional panic episodes.
- Primary (functional) childhood pain disorders, including growing pains and headaches in early childhood, and a diagnosis of common migraine several years later.
- Iron deficiency, though it was unknown whether this dated from infancy.
- Trauma resulting in fracture of radius and ulna at 8 years of age and fracture of an ankle a year or two later without major sequelae.
- Motor vehicle accident with cervical whiplash injury at 9 years of age.
- Hypermobility of joints.
- Severe *Streptococcal* throat infection at 16 years of age, followed by chronic fatigue from which she had not recovered. This coincided with the exacerbation and chronicity of her pain.

In view of the array of risk markers combining to create a high-risk phenotype for chronic pain disorders, consideration was given as to why the expression of this young woman's chronic pain disorder was relatively mild. It was apparent that she possessed protective factors including emotional intelligence, social support, good employment, and regular exercise.

## 2. Methods

A literature review was performed by searching the electronic bibliographic databases MEDLINE (via Ovid) and EMBASE (via Ovid), using strategies widely used in systematic reviews, from their inception until September 2017. Search terminology was identified through the controlled vocabulary thesauruses Medical Subject Headings (MeSH) and Emtree. To identify the most appropriate MeSH, the MeSH tree structure was used, and the subject headings “chronic pain,” “fibromyalgia,” “risk factors,” “child,” “adolescent,” and “young adult” were selected. This method was replicated for Emtree, and identical subject headings were selected. The search was limited to English language publications, but no other filters were applied. Subject headings were combined with the Boolean operators “OR” and “AND” to find their intersection (Table 1).

Citations were screened by title and abstract for relevance to risk markers for CWP or FMS in children, adolescents, or young adults. The full-text publications of relevant abstracts were attained, and the reference lists of relevant studies were manually reviewed. Our primary focus was family, genetic, and pediatric association studies. Studies of risk markers for CWP or FMS in adults were added when it was apparent that many risk markers provide inferences and hypotheses applicable to young adults and adolescents. Studies of associations and comorbidities likely to be secondary to CWP or FMS were excluded, including restless legs syndrome, low bone mineral density, and cardiovascular disease aggravation. Trauma and infection were included because, like most risk markers, they typically have only contributory causal influences.

The literature search identified 475 articles. After excluding 93 duplicates, citation screening of the remaining 382 articles excluded 276 articles based on irrelevance. The full-text publications of the remaining 106 articles were obtained, reviewed, and qualitatively synthesized. To grade the strength of the research evidence, we adapted the modified grading of recommendations assessment, development, and evaluation (GRADE) framework for a narrative synthesis by Huguet et al. [36].

## 3. Results

Table 2 presents the studies of familial and genetic influences on CWP and FMS. Until recently, FMS diagnosis required physical examination, and thus, large population studies were unavailable. Stronger evidence for familial and genetic risk markers for CWP has come from population-based twin studies.

Table 3 lists the published risk markers for CWP and FMS determined in pediatric studies. These risk markers reflect underlying neurobiological processes which are incompletely understood. Overall, the graded strength of research evidence, determined using the four quality categories defined by the original GRADE framework [65], high, moderate, low, and very low quality, was greater for risk markers for CWP than FMS because of the earlier availability of questionnaire surveys.

Table 4 displays the modifiable and nonmodifiable risk markers for CWP and FMS from adult studies. Generally, the data were applicable to young adults and adolescents. Again, stronger evidence for risk markers applied to CWP.

### 3.1. Familial and Genetic Vulnerability

In a population-based twin study [39], Vehof et al. demonstrated that chronic pain syndromes, including CWP, irritable bowel syndrome, chronic pelvic pain, and spinal pain, [40] shared an underlying genetic factor with an estimated heritability of 66%. Vehof and Williams [93] suggested that the once evolutionary advantage of high pain sensitivity may have turned into a genetic predisposition to the neurobiological underpinnings of chronic pain. Parental CWP was shown to increase the risk of CWP in adult offspring, particularly if both parents had CWP, and offspring were obese [41]. Reviewing eight twin studies on CWP, six molecular genetic studies on CWP, and one epigenetic study, the findings by Kerr and Burri [42] suggested that genetic and unique environmental factors contributed to CWP. Various candidate genes, such as serotonin-related pathway genes, were found to be associated with CWP.

Most studies which have documented psychosocial determinants of CWP have been unable to exclude genetic confounding, given the strong heritability of some risk markers, notably depression and anxiety. Burri et al. [71] used a discordant monozygotic-twin approach to overcome the traditional limitations of conventional epidemiology by separating environmental influences from genetic confounders to provide clues on the direction of causality. The results, supported by another twin study [38], demonstrated that the covariance between CWP and depression and anxiety was due to a common, highly heritable, and latent trait.

Research on genetic predisposition to FMS has revealed associations between genetic factors and FMS, including specific gene polymorphisms involved in serotonergic, dopaminergic, and catecholaminergic pathways [44, 94]. FMS coaggregated in families with reduced pressure pain thresholds and major mood disorder [43].

### 3.2. Female Sex

CWP and FMS demonstrated female predominance [95], with juvenile FMS affecting females four times more than males [29]. This female predominance is incompletely understood, but likely involves genetic, psychological, behavioral (including reporting behavior), and neurobiological factors [68].

### 3.3. Early-Life Adversity

Early-life adversity was a risk marker for developing adult CWP and FMS. Epidemiological studies of the 1958 British Birth Cohort Study demonstrated that multiple somatic symptoms at age 7 were independently associated with CWP at age 45 [19, 45, 46]. Moderate associations included headaches, abdominal pain, and periodic vomiting [46], and significant associations included hospitalization following a road traffic accident, residence in institutional care, maternal death, familial financial hardship, and persistent behavioral problems [45, 76, 96]. Whilst the association between preterm birth and very low birthweight and adult CWP was not statistically significant [77], extremely premature babies who underwent repeated procedures after birth demonstrated sustained changes in sensory processing by age 11 [97].

Other studies have demonstrated that painful infant experiences and childhood maltreatment were associated with adult FMS. These associations included preterm birth, perinatal exposure to substances of abuse, maternal deprivation, growing up with a depressed parent, psychological trauma, physical or verbal abuse from parents, and physical or sexual abuse by an adult [47–49]. Abuse in childhood was associated with a 97% increased risk of painful somatic syndromes in adulthood, including FMS [98].

Early-life adversity may increase vulnerability to CWP and FMS by excessively activating stress responses during a critical period and altering normal development, stress reactivity, and nociceptive function through hyperalgesic priming. The pain itself is a stressor and, as it evolves, may generate a positive feedback loop to increase anxiety levels and impact stress regulation.

### 3.4. Cognitive and Psychosocial Factors

Early childhood cognitive and psychosocial problems are risk markers for CWP. In children, the independent risk markers for widespread pain were adverse behavioral and emotional factors, somatic pain symptoms, older age, female sex, depression, and regional back and neck pain symptoms [19, 20]. In adolescents, CWP was associated with anxiety, depression, and conduct or attentional problems [21]. For young people aged 10–18, higher widespread pain scores were associated with poorer mental and physical health-related quality of life and greater functional impairment. Whilst this relationship was explained by psychosocial distress, including somatization features, health-seeking behavior, insecure attachment style, and poor sleep, the causal direction has not been established [52, 54, 70, 99]. Resilience factors, including high self-esteem, seldom feeling lonely, and high scores for family cohesion or social confidence, were associated with a lower prevalence, and markedly attenuated the association between psychiatric symptoms and CWP [21].

In adults, prospective studies from the 1958 British Birth Cohort Study demonstrated that low socioeconomic status at age 7 was associated with CWP at age 45 [60] and appeared to be explained by psychological factors [52]. Additionally, low intellectual and emotional intelligence, low social class, low educational attainment, high body mass index, and psychological distress in childhood were independent predictors of CWP in adulthood [51].

In later life, risk markers for CWP included the psychological factors of anxiety and depression, lifestyle factors of smoking and obesity, and other factors such as dysfunction of the hypothalamic-pituitary-adrenal axis [66, 89]. Posttraumatic stress disorder was strongly associated with CWP and FMS [59, 73, 74], likely mediated in part by the known enhanced influence on central sensitization and common antecedent traumatic experiences.

Juvenile FMS was associated with temperamental instability, low mood, depression and anxiety, family disorder, social isolation, higher pain and emotional sensitivity, and lower psychological adjustment [53, 58]. Psychiatric disorders, notably anxiety, were prevalent in juvenile and early adulthood FMS and were associated with greater physical impairment, poorer functioning, and lower health-related quality of life [56, 57]. The lifetime prevalence of psychiatric comorbidity, notably depression, anxiety, and bipolar disorder, with FMS may suggest a shared underlying pathophysiological link.

### 3.5. Sleep Problems

Poor self-reported sleep quality was a risk marker for CWP and FMS [78]. Whilst children with juvenile FMS demonstrated abnormal *α*-rhythms (suggestive of wakefulness during nonrapid eye movement sleep), periodic limb movements, prolonged sleep latency, shortened total sleep time, decreased sleep efficiency, and increased wakefulness during sleep, the causal relationship is unclear [61]. Sleep deprivation impairs descending pain-inhibition pathways and can cause myalgia, tenderness, and fatigue, suggesting that sleep dysfunction might not only be a consequence of pain but also pathogenetic [78].

### 3.6. Physical Trauma

The 1958 British Birth Cohort Study demonstrated the association between childhood trauma and CWP [45]. One of the major childhood risk markers for adult CWP was hospitalization following a road traffic accident, though this may be influenced by the associated psychological sequalae.

Of individuals who presented to an emergency department after motor vehicle collision, 11% developed CWP [79]. This is consistent with evidence that spinal pain not uncommonly evolves into CWP. In adult females with FMS, childhood trauma had a clinically important association with loss of functionality and comorbid depression [100].

### 3.7. Functional/Primary Pain Disorders and Multiregional Pain Disorders

CWP and FMS often evolved over time from multiregional pain disorders. Headaches and abdominal pain in children were associated with a doubling of the risk of developing widespread body pain in the ensuing year [19]. Children aged 7 with abdominal pain, headaches, migraine, or vomiting attacks had an increased risk of CWP at age 45 [46].

Adults with back or neck pain were particularly at risk of CWP, and the same risk was likely to apply to children and adolescents [15]. The incidence of CWP in adults with chronic low back pain was 23.8% [80], and the rate of developing CWP within 5-6 years in adults with regional back or neck pain was 22.6% [81]. The risk markers associated with the transition to CWP were moderate or severe pain intensity, female sex, long duration of back pain, high rate of psychosomatic symptoms, history of abuse, family history of CWP, severe interference with general activities, having one or more central sensitivity syndromes, and using more pain management strategies. Additionally, functional somatic syndromes were risk markers for CWP, specifically chronic fatigue, irritable bowel syndrome, and chronic pelvic pain [82], and for FMS, specifically migraine [83, 84].

### 3.8. Hypermobility of Joints

In adolescents with juvenile FMS, 48% have been clinically assessed as having hypermobility of joints [62]. Hypermobility of joints was associated with enhanced physiological pain sensitivity.

### 3.9. Obesity

Obesity and low physical activity have been epidemiologically associated with chronic pain, including CWP [66, 86] and FMS [86, 101]; however, studies in adolescents have only reported associations between obesity and the prevalence and severity of knee pain and chronic regional pain [102]. Obesity can be considered an aggravating comorbid condition affecting condition severity, quality of life, fatigue, and physical dysfunction. Poor physical fitness was a key covariate in increasing the risk of both widespread pain and obesity [85]. Other potential causal influences of obesity on CWP and FMS included physical inactivity, sleep disturbance, depression, endocrine dysfunction, lower pressure pain thresholds, and inflammatory and immune effects [103–105].

### 3.10. Rheumatic Conditions

The 2016 revised fibromyalgia criteria permitted a diagnosis of FMS in association with any medical illness, including inflammatory musculoskeletal disorders. There has been a tendency for CWP and a deep hyperalgesic state occurring in 10–30% of patients with primary rheumatic diseases, including rheumatoid arthritis and systemic lupus erythematosus, to be termed secondary or comorbid FMS. However, this is best referred to by the primary diagnosis and the additional interpretation of widespread deep secondary allodynia or low-pressure pain threshold reflecting central sensitization [95]. It is acknowledged that the inflammatory rheumatic condition might not be the sole source of nociceptive input or source of central somatosensory dysfunction.

### 3.11. Iron Deficiency

Iron deficiency, especially in early life, may be a risk marker for CWP. In a twin family study, we observed an independent association between iron deficiency and chronic pediatric pain [106]. A serum ferritin level <50 ng/ml was associated with a 6.5-fold increased risk for FMS in females [87]. Paradoxically, FMS has also been associated with hemochromatosis [88].

### 3.12. Infectious Illness

Various infections have been linked to FMS. Epstein–Barr and parvovirus infections, brucellosis, and Lyme disease were often cited [90, 91], but any infectious illness characterized by prolonged bed rest and persistent fatigue might be a causal influence. Postinfection chronic fatigue syndrome is probably a more appropriate diagnostic term, especially in those in whom fatigue is dominant over pain. Microglial activation may underpin postinfectious contributions to CWP.

### 3.13. Small-Fiber Polyneuropathy

Small-fiber polyneuropathy, alongside chronic neuroinflammation, was associated with CWP and FMS, with claims of causation. In patients with widespread pain beginning before age 21, small-fiber polyneuropathy was “definite” in 59% and “probable” in 17% [64].

## 4. Discussion

The literature leaves little doubt that some children and adolescents have increased vulnerability to developing CWP and FMS. The risk markers for CWP and FMS suggest a complex etiological structure which shares risk markers with those identified in other chronic pain disorders. Evidence for potential mechanisms underpinning these risk markers is emerging at molecular, cellular, and network levels [107]. Alterations in the structure, wiring, function, and neurochemistry of various brain networks, particularly the reward-motivation network and the endogenous pain modulation system, might be involved in conferring vulnerability to pain conditions. Epigenetic modulation, leading to changes to neuronal and molecular processes, is one way in which genetics and adverse priming events, including prior injury or stressful environmental influences, may increase risk. In FMS, neuropathological changes include regional changes in grey matter volume, decreased functional connectivity in the descending pain-modulating system, and an increased activity in the pain matrix related to central sensitization. Whether measurable alterations in brain function precede or follow the onset of chronic pain, they might lead to a vicious cycle in which vulnerability leads to nonresilience to additional factors arising from the chronic pain state. It is likely that biological, psychological, social, and contextual risk markers interact to predispose, initiate, maintain, and exacerbate CWP and FMS [72].

**Figure 1:**
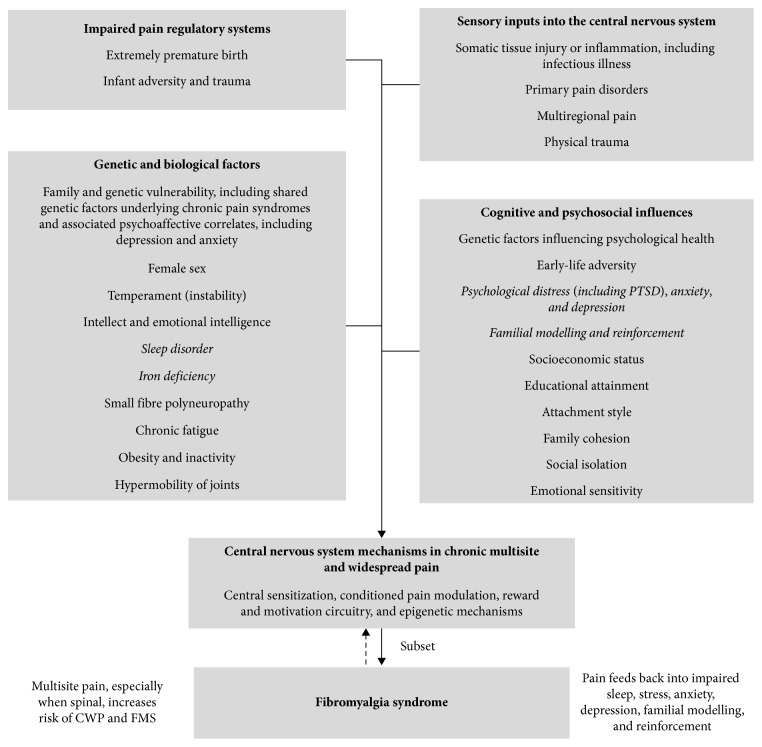
Biopsychosocial model of risk markers for chronic widespread pain and fibromyalgia syndrome in young people. *Note.* Risk markers for which there is limited evidence have been italicized.

A model of life-course influences on the development of chronic pain was published by Dominick and Blythe [108]. The model by Von Baeyer and Champion [12] was developed to illustrate factors in the development of multiple functional (or primary) pain disorders, but could appropriately be applied to life-course risk markers in the development of CWP and FMS in young people. It is likely that biological, psychological, social, and contextual variables interact to influence the predisposition, triggering, and aggravation of CWP and FMS. To integrate current knowledge, theory, and hypotheses, we propose a biopsychosocial model of risk markers for chronic widespread pain and fibromyalgia syndrome in young people (Figure 1). For a more specific model for FMS, see Häuser et al. [91], and for juvenile FMS, see Kashikar-Zuck and Ting [28].

The majority of the risk markers have at least some potential for modification. There is less or no prospect of favorable modification of sex, temperament, intelligence, hypermobility of joints, small-fiber polyneuropathy, physical trauma, and chronic fatigue.

Although this review was based on extensive literature review and evaluation of the strength of research evidence, the review was limited by the possible selectivity of searches and citations, and publication bias underestimating negative results.

## 5. Conclusion

The strongest evidence for risk markers as potential causal influences on CWP and FMS (Tables 2–4) came from prospective cohort studies, notably birth cohorts, and genetic studies, mainly twin analyses. Genetic influences were prominent but not always overt, including shared genes between widespread pain and depression, widespread pain and spinal pain, and functional/primary pain syndromes. Low emotional intelligence merits more attention, given the strength of evidence. The social risk markers, including childhood adversity and low socioeconomic status, were prominent across numerous studies and were at least partly related to stress, anxiety, and depression.

The case presented illustrates how a history of risk markers over the life-course can provide insight into why a young person might acquire CWP fulfilling criteria for FMS. Her risk markers were family history, premature birth, childhood adversity, hypermobility of joints, mild obesity, anxiety and depression, multiple primary or functional pain disorders, physical trauma, iron deficiency, and infectious illness with chronic fatigue. Such a cumulative burden evidently determined the onset by adolescence of her CWP, but typically the diagnostic categorization was made several years after the symptoms had become well established, notwithstanding consultations and intensive investigations. The taking of an earlier insightful history of the risk markers could have identified reversible factors leading to a more favorable trajectory and might have worked to mutual advantage for the patient and physician. It is likely that personal resilience can limit the severity of CWP and FMS and warrants further research with an aim to developing interventions designed to increase personal resilience.

## Figures and Tables

**Table 1:** Search strategy.

#	Searches	Results
MEDLINE		
1	Exp chronic pain/	9400
2	Exp fibromyalgia/	7898
3	1 or 2	16903
4	Exp risk factors/	730540
5	Exp child/	1810174
6	Exp adolescent/	1905521
7	Exp young adult/	646590
8	5 or 6 or 7	3134864
9	3 and 4 and 8	240
10	Limit 9 to English language	230

EMBASE		
1	Exp chronic pain/	48537
2	Exp fibromyalgia/	17126
3	1 or 2	62529
4	Exp risk factor/	837344
5	Child/	1681331
6	Adolescent/	1453055
7	Exp young adult/	200970
8	5 or 6 or 7	2611330
9	3 and 4 and 8	255
10	Limit 9 to English language	245

Initial number of articles	475	
After excluding duplicate articles	382	
After excluding irrelevant articles	106	
Final number of articles	106	

**Table 2:** Familial and genetic influences for chronic widespread pain and fibromyalgia.

Pain context	Type of study	Population or clinic	Number of participants	Multivariate analysis	Reference	Overall quality^*∗*^
CWP	Twin study	Population	44,897	No	Kato et al. [37]	+++
CWP	Twin study	Population	926	Yes	Burri et al. [38]	++++
CWP	Twin study	Population	8564	Yes	Vehof et al. [39]	+++
CWP	Twin study	Population	2256	Yes	Malkin et al. [40]	+++
CWP	Prospective population cohort	Population	6589	Yes	Zadro et al. [41]	++++
CWP	Review	N/A	N/A	N/A	Kerr and Burri [42]	N/A
FMS	Family study	Clinic	805	Yes	Arnold et al. [43]	++
FMS	Review	N/A	N/A	N/A	Park et al. [44]	N/A

^*∗*^Overall quality:  + , very low;  ++ , low;  +++ , moderate;  ++++ , high.

**Table 3:** Risk markers for chronic widespread pain and fibromyalgia in pediatric samples.

Risk marker	Modifiable	Pain context	Type of study	Population or clinic	Number of participants	Multivariate analysis	Reference	Overall quality^*∗*^
Female sex	No	CWP	Prospective population cohort	Population	1282	Yes	Mikkelsson et al. [20]	+++

Early-life adversity	Possibly	CWP	Prospective population cohort	Population	7,571	Yes	Jones et al. [19, 45, 46]	+++
		CWP	Prospective population cohort	Population	7,470	No	Jones et al. [19, 45, 46]	+++
		CWP	Prospective population cohort	Population	1,440	Yes	Jones et al. [19, 45, 46]	+++
		FMS	Case-control	Clinic	36	No	Olivieri et al. [47]	+
		FMS	Case-control	Clinic	117	No	Bohn et al. [48]	+
		FMS	Review	N/A	N/A	N/A	Low and Schweinhardt [49]	N/A
		FMS	Case-control retrospective	Clinic	75	Yes	Hellou et al. [50]	++

*Psychosocial factors*								
(i) Intelligence (low)	No	CWP	Population survey	Population	6,902	Yes	Gale et al. [51]	+++
(ii) Insecure attachment	Possibly	CWP	Prospective population cohort	Population	2,509	No	Davies et al. [52]	+++
(iii) Peer relationships	Yes	FMS	Case-control	Clinic	110	No	Kashikar-Zuck et al. [53]	+
(iv) Stress, anxiety, and depression	Yes	CWP	Prospective population cohort	Population	7,571	Yes	Jones et al. [19, 45, 46]	+++
		CWP	Prospective population cohort	Population	7,470	Yes	Jones et al. [19, 45, 46]	+++
		CWP	Prospective population cohort	Population	1,440	Yes	Jones et al. [19, 45, 46]	+++
		CWP	Prospective population cohort	Population	2,650	Yes	Nicholl et al. [54]	+++
		CWP	Cross-sectional	Population	7,070	No	Skrove et al. [21]	++
		FMS	Population survey	Population	3,006	Yes	Wolfe et al. [55]	++
		FMS	Case series	Clinic	76	No	Kashikar-Zuck et al. [56]	+
		FMS	Prospective cohort	Clinic	121	Yes	Cunningham et al. [57]	+++
(v) Temperament	Possibly	FMS	Case-control	Clinic	48	No	Conte et al. [58]	+
(vi) Posttraumatic stress disorder	Possibly	CWP	Cross-sectional survey	Population	3,740	No	Arguelles et al. [59]	++
(vii) Socioeconomic status (low)	Possibly	CWP	Prospective population cohort	Population	9,377	Yes	Macfarlane et al. [60]	+++
Sleep problems	Yes	FMS	Case series	Clinic	30	No	Tayag-Kier et al. [61]	+
Physical trauma	No	CWP	Prospective population cohort	Population	7,571	Yes	Jones et al. [45]	+++
Functional/primary pain disorders	Yes	CWP	Prospective population cohort	Population	7,470	No	Jones et al. [19, 46]	+++
		CWP	Prospective population cohort	Population	1,440	Yes	Jones et al. [19, 46]	+++
Hypermobility of joints	No	FMS	Cross-sectional	Clinic	131	No	Ting et al. [62]	+
Chronic fatigue	No	CWP	Population birth cohort	Population	3214	Yes	Norris et al. [63]	++++
Small-fiber polyneuropathy	No	FMS	Case series	Clinic	41	No	Oaklander and Klein [64]	+

^*∗*^Overall quality:  + , very low;  ++ , low;  +++ , moderate;  ++++ , high.

**Table 4:** Risk markers for chronic widespread pain and fibromyalgia in adult samples.

Risk marker	Modifiable	Pain context	Type of study	Population or clinic	Number of participants	Multivariate analysis	Reference	Overall quality^*∗*^
Female sex	No	CWP	Prospective population cohort	Population	28,367	Yes	Mundal et al. [66]	+++
		CWP	Systematic review	N/A	N/A	N/A	Mansfield et al. [67]	N/A
		CWP	Review	N/A	N/A	N/A	Mogil [68]	N/A
		FMS	Random population survey	Population	830	No	Vincent et al. [69]	++

*Psychosocial factors*								
(i) Stress, anxiety, and depression	Yes	CWP	Prospective population cohort	Population	28,367	Yes	Mundal et al. [66]	+++
		CWP	Prospective population cohort	Population	3,171	Yes	Gupta et al. [70]	+++
		CWP	Twin survey	Population Population Population	3,266 9,26 3,266	Yes Yes Yes	Burri et al. [38, 71, 72]	++++
(ii) Emotional intelligence (low)	Possibly	CWP	Twin survey	Population	3,266	Yes	Burri et al. [71]	++++
(iii) Posttraumatic stress disorder	Possibly	CWP	Prospective population cohort	Population	1,312	Yes	Raphael et al. [73]	+++
		FMS	Cross-sectional	Clinic	395	No	Häuser et al. [74]	++
(iv) Socioeconomic status (low)	Possibly	CWP	Prospective population cohort	Population	2,509	No	Davies et al. [52]	+++
(v) Family dysfunction	Possible	CWP	Case-control cross-sectional	Clinic	75	Yes	Hayaki et al. [75]	++
(vi) Maladjusted behavior	Yes	CWP	Prospective population cohort	Population	8572	Yes	Pang et al. [76]	+++

Preterm birth	No	CWP	Prospective population cohort	Population	8572	Yes	Littlejohn et al. [77]	+++

Sleep problems	Yes	CWP	Prospective population cohort	Population	3,171	Yes	Gupta et al. [70]	+++
		FMS	Review	N/A	N/A	N/A	Choy [78]	N/A

Physical trauma	No	CWP	Prospective cohort	Clinic	948	No	Hu et al. [79]	++

Functional/primary pain disorders	Yes	CWP	Prospective cohort	Clinic	423	Yes	Viniol et al. [80]	++
		CWP	Retrospective cohort	Clinic	512	Yes	Kindler et al. [81]	++
		FMS	Case-control	Clinic	625	Yes	Warren et al. [82]	++
		FMS	Cross-sectional	Clinic	1,123	Yes	de Tommaso et al. [83]	++
		FMS	Controlled trial	Clinic	86	No	Giamberardino et al. [84]	+

Overweight and obesity	Yes	CWP	Prospective population cohort	Population	28,367	Yes	Mundal et al. [66]	+++
		CWP	Prospective population cohort	Population	1,553	Yes	Magnusson et al. [85]	+++
		FMS	Review	N/A	N/A	N/A	Ursini et al. [86]	N/A

Iron deficiency	Yes	FMS	Case-control	Clinic	92	No	Ortancil et al. [87]	+

Hemochromatosis	Yes	FMS	Cross-sectional	Clinic	295	No	Mohammad et al. [88]	+

HPA dysfunction	Possibly	CWP	Prospective population cohort	Population	241	Yes	McBeth et al. [89]	+++

Infectious illness	Possibly	FMS	Review	N/A	N/A	N/A	Buskila et al. [90]	N/A
		FMS	Review	N/A	N/A	N/A	Häuser et al. [91]	N/A
		FMS	Prospective cohort controlled	Population	1,244	Yes	Chen et al. [92]	+++

^*∗*^Overall quality:  + , very low;  ++ , low;  +++ , moderate;  ++++ , high.

## Abbreviations

CWP: Chronic widespread pain
FMS: Fibromyalgia syndrome.
